# Complete genome sequence of *Veillonella parvula* type strain (Te3^T^)

**DOI:** 10.4056/sigs.521107

**Published:** 2010-01-28

**Authors:** Sabine Gronow, Sabine Welnitz, Alla Lapidus, Matt Nolan, Natalia Ivanova, Tijana Glavina Del Rio, Alex Copeland, Feng Chen, Hope Tice, Sam Pitluck, Jan-Fang Cheng, Elizabeth Saunders, Thomas Brettin, Cliff Han, John C. Detter, David Bruce, Lynne Goodwin, Miriam Land, Loren Hauser, Yun-Juan Chang, Cynthia D. Jeffries, Amrita Pati, Konstantinos Mavromatis, Natalia Mikhailova, Amy Chen, Krishna Palaniappan, Patrick Chain, Manfred Rohde, Markus Göker, Jim Bristow, Jonathan A. Eisen, Victor Markowitz, Philip Hugenholtz, Nikos C. Kyrpides, Hans-Peter Klenk, Susan Lucas

**Affiliations:** 1 DSMZ – German Collection of Microorganisms and Cell Cultures GmbH, Braunschweig, Germany; 2 DOE Joint Genome Institute, Walnut Creek, California, USA; 3 Los Alamos National Laboratory, Bioscience Division, Los Alamos, New Mexico, USA; 4 Oak Ridge National Laboratory, Oak Ridge, Tennessee, USA; 5 Biological Data Management and Technology Center, Lawrence Berkeley National Laboratory, Berkeley, California, USA; 6 HZI – Helmholtz Centre for Infection Research, Braunschweig, Germany; 7 University of California Davis Genome Center, Davis, California, USA

**Keywords:** opportunistic infections, human oral microflora, dental plaque, intergeneric coaggregation, methylmalonyl-CoA decarboxylase, *Veillonellaceae*

## Abstract

*Veillonella parvula* (Veillon and Zuber 1898) Prévot 1933 is the type species of the genus *Veillonella* in the family *Veillonellaceae* within the order *Clostridiales*. The species *V. parvula* is of interest because it is frequently isolated from dental plaque in the human oral cavity and can cause opportunistic infections. The species is strictly anaerobic and grows as small cocci which usually occur in pairs. *Veillonellae* are characterized by their unusual metabolism which is centered on the activity of the enzyme methylmalonyl-CoA decarboxylase. Strain Te3^T^, the type strain of the species, was isolated from the human intestinal tract. Here we describe the features of this organism, together with the complete genome sequence, and annotation. This is the first complete genome sequence of a member of the large clostridial family *Veillonellaceae*, and the 2,132,142 bp long single replicon genome with its 1,859 protein-coding and 61 RNA genes is part of the *** G****enomic* *** E****ncyclopedia of* *** B****acteria and* *** A****rchaea * project.

## Introduction

Strain Prévot Te3^T^ (= DSM 2008 = ATCC 10790 = JCM 12972) is the type strain of the species *Veillonella parvula* and was first described in 1898 by Veillon and Zuber [1] as “*Staphylococcus parvulus*” before it was renamed as *Veillonella parvula* by Prévot in 1933 [2]. Although it is a Gram-negative organism harboring lipopolysaccharide [3] it is more closely related to Gram-positive species like *Sporomusa*, *Megasphaera* or *Selenomonas*. Together, they share the unusual presence of cadaverine and putrescine in their cell walls [4]. The genus *Veillonella* comprises 11 species (status July 2009) which are all known to inhabit the oral cavity and the gastrointestinal tract of homeothermic vertebrates. Six of the species, among them *V. parvula*, have been isolated from man, the others are typical for rodents [5]. In general, veillonellae are harmless inhabitants of most body cavities, however, occasionally they can participate in multispecies infections at diverse body sites and in rare cases cause severe infections also as pure cultures [6].

Here we present a summary classification and a set of features for *V. parvula* Te3^T^, together with the description of the complete genomic sequencing and annotation.

## Classification and features

The natural habitat is human dental plaque and *V. parvula* can amount to up to 98% of the cultivable veillonellae in healthy subgingival sites [7]. Additionally, veillonellae are common inhabitants of the gastrointestinal tract. Although the other species of the genus *Veillonella* are found in large numbers throughout the oral cavity, *V. parvula* is the only species of the genus involved in oral diseases such as gingivitis. It has also been isolated in rare cases of endocarditis, meningitis, discitis [8] or bacteremia as pure culture but more often *V. parvula* is involved in multispecies infections (reviewed in [6]). Medline indexes few cultivated strains with a high degree of 16S rRNA gene sequence similarity to Te3^T^, e.g. DJF_B315 from porcine intestine (EU728725, Hojberg and Jensen, unpublished, 99.9% identity). The other type strains of the genus *Veillonella* vary from 94.1% (*V. ratti*) to 99.2% (*V. dispar*). A vast number of phylotypes with significant 16S rRNA sequence similarity to *V. parvula* were observed from intubated patients [9], carious dentine from advanced caries (AY995757; 99.7% identity), and the human skin microbiome [10]. Curiously, only one sample from a human gut metagenome analysis [11] scored above 96% sequence similarity in screenings of environmental samples (status September 2009).

**Figure 1:**
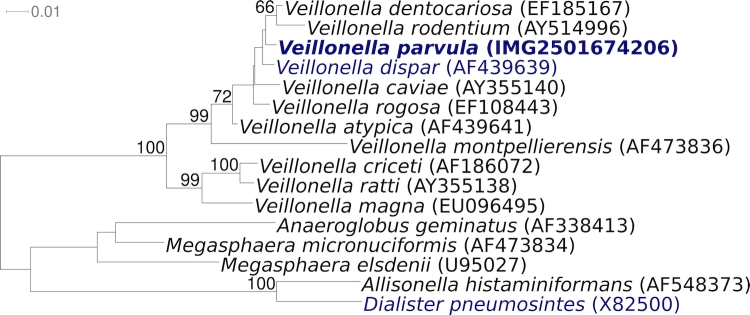
Phylogenetic tree highlighting the position of *V. parvula* strain Te3^T^ relative to all other type strains within the genus *Veillonella*. The tree was inferred from 1,378 aligned characters [12,13] of the 16S rRNA gene sequence under the maximum likelihood criterion [14]. The tree was rooted with the type strains of other genera within the family *Veillonellaceae*. The branches are scaled in terms of the expected number of substitutions per site. Numbers above branches are support values from 1,000 bootstrap replicates if greater than 60%. Lineages with type strain genome sequencing projects registered in GOLD [15] are shown in blue, published genomes in bold.

Figure 1 shows the phylogenetic neighborhood of *V. parvula* strain Te3^T^ in a 16S rRNA based tree. The sequences of the four copies of the 16S rRNA gene in the genome differ by up to seven nucleotides, and differ by up to four nucleotides from the previously published sequence generated from ATCC 10790 (AY995767).

**Figure 2:**
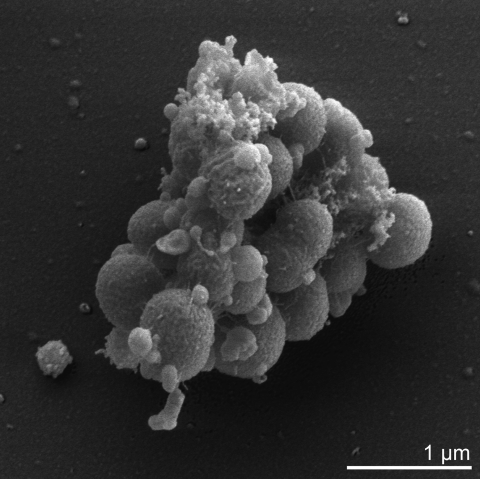
Scanning electron micrograph of *V. parvula* Te3^T^

*V. parvula* is a Gram-negative, non-motile, non-sporeforming, anaerobic coccus (approximately 0.3 to 0.5 µm in diameter) that grows in pairs or short chains (Table 1 and Figure 2). Veillonellae are characterized by an unusual metabolism using methylmalonyl-CoA decarboxylase to convert the free energy derived from decarboxylation reactions into an electrochemical gradient of sodium ions [29]. They utilize the metabolic end products of co-existing carbohydrate-fermenting bacteria, i.e. lactic acid bacteria in the gastrointestinal tract, and thereby play an important role in a natural microbial food chain [6]. Another characteristic trait of veillonellae is their ability to form intergeneric coaggregates with other bacteria which occur in the same ecological niche [30]. Although *Veillonella* cannot adhere to surfaces itself, the bacterium is able to attach to specific surface structures present on other cells, often mediated by lectin-carbohydrate interactions [31]. The coaggregation creates a functional community providing nutrients and protection for all participants.

**Table 1:** Classification and general features of *V. parvula* Te3^T^ according to the MIGS recommendations [16].

**MIGS ID**	**Property**	**Term**	**Evidence code**
	Current classification	Domain *Bacteria*	TAS [17]
		Phylum *Firmicutes*	TAS [18,19]
		Class *Clostridia*	TAS [18]
		Order *Clostridiales*	TAS [20,21]
		Family *Veillonellaceae*	TAS [22,23]
		Genus *Veillonella*	TAS [2]
		Species *Veillonella parvula*	TAS [2]
		Type strain Te3	TAS [2]
	Gram stain	negative	TAS [1]
	Cell shape	small cocci	TAS [1]
	Motility	nonmotile	TAS [1]
	Sporulation	nonsporulating	TAS [1]
	Temperature range	mesophile	TAS [1]
	Optimum temperature	37°C	TAS [1]
	Salinity	normal	TAS [1]
MIGS-22	Oxygen requirement	anaerobic	TAS [1]
	Carbon source	acid production from lactate and other organic acids	TAS [24]
	Energy source	lactate; succinate	TAS [25,26]
MIGS-6	Habitat	human gastrointestinal tract	TAS [1]
MIGS-15	Biotic relationship	human pathogen	TAS [1]
MIGS-14	Pathogenicity	subgingival plaque formation; opportunistic pathogen	TAS [1]
	Biosafety level	2	TAS [27]
	Isolation	human intestinal tract	TAS [1]
MIGS-4	Geographic location	unknown, probably France	TAS [1]
MIGS-5	Sample collection time	before 1898	TAS [1]
MIGS-4.1 MIGS-4.2	Latitude, Longitude	unknown	
MIGS-4.3	Depth	not reported	
MIGS-4.4	Altitude	not reported	

Evidence codes - IDA: Inferred from Direct Assay (first time in publication); TAS: Traceable Author Statement (i.e., a direct report exists in the literature); NAS: Non-traceable Author Statement (i.e., not directly observed for the living, isolated sample, but based on a generally accepted property for the species, or anecdotal evidence). These evidence codes are from of the Gene Ontology project [28]. If the evidence code is IDA, then the property was observed for a live isolate by one of the authors or an expert mentioned in the acknowledgements.

Strain Te3^T^ produces propionic and acetic acid, carbon dioxide and hydrogen from lactate and other organic acids like pyruvate, malate or fumarate. *V. parvula* cannot grow on succinate as a sole carbon source but can decarboxylate succinate during fermentation of lactate or malate [25]. Veillonellae are unable to use glucose or other carbohydrates for fermentation [26] and they do not possess a functional hexokinase [24]. Nitrate is reduced and arginine dihydrolase is produced.

Veillonellae show resistance to tetracycline (>25 µg/ml), erythromycin (>25 µg/ml) gentamicin (>25 µg/ml) and kanamycin (>25 µg/ml) and they are susceptible to penicillin G (0.4 µg/ml), cephalotin (1.6 µg/ml) and clindamycin (0.1 µg/ml). Their resistance is intermediate for chloramphenicol (3.1 µg/ml) and lincomycin (6.2 µg/ml) [32].

### Chemotaxonomy

The cell wall of *V. parvula* comprises an outer membrane, clearly demonstrating the presence of lipopolysaccharide [33]. The peptidoglycan of veillonellae is of the A1γ-type with glutamic acid in D configuration, diaminopimelic acid in meso configuration and covalently bound cadaverine or putrescine attached in α-linkage to glutamic acid [34]. As major fatty acids straight-chain saturated C_13:0_ (24%), C_15:0_ (12%) and C_16:0_ (7%) and unsaturated C_16:1_ (5%), C_17:1_ (22%) and C_18:1_ (6%) are synthesized [35]. Another characteristic feature of *V. parvula* is the presence of plasmalogens such as plasmenylethanolamine and plasmenylserine as major constituents of the cytoplasmic membrane. These ether lipids replace phospholipids and play an important role in the regulation of membrane fluidity [36].

## Genome sequencing and annotation

### Genome project history

This organism was selected for sequencing on the basis of its phylogenetic position, and is part of the *** G****enomic* *** E****ncyclopedia of* *** B****acteria and* *** A****rchaea * project. The genome project is deposited in the Genome OnLine Database [15] and the complete genome sequence is deposited in GenBank. Sequencing, finishing and annotation were performed by the DOE Joint Genome Institute (JGI). A summary of the project information is shown in Table 2.

**Table 2:** Genome sequencing project information

**MIGS ID**	**Property**	**Term**
MIGS-31	Finishing quality	Finished
MIGS-28	Libraries used	Two Sanger libraries: 8kb pMCL200 and fosmid pcc1Fos One 454 pyrosequence standard library
MIGS-29	Sequencing platforms	ABI3730, 454 GS FLX
MIGS-31.2	Sequencing coverage	7.67× Sanger; 50.6× pyrosequence
MIGS-30	Assemblers	Newbler, Phrap
MIGS-32	Gene calling method	Prodigal, GenePRIMP
	INSDC ID	CP001820
	Genbank Date of Release	23-November 2009
	GOLD ID	Gc01152
	NCBI project ID	21091
	Database: IMG-GEBA	2501651195
MIGS-13	Source material identifier	DSM 2008
	Project relevance	Tree of Life, GEBA

### Growth conditions and DNA isolation

*V. parvula* strain Te3^T^, DSM 2008, was grown anaerobically in DSMZ medium 104 (modified PYG-Medium, with the addition of lactate and putrescine) at 37°C [37]. DNA was isolated from 1.5-2 g of cell paste using Qiagen Genomic 500 DNA Kit (Qiagen, Hilden, Germany) following the manufacturer’s protocol, with cell lysis protocol L as described in Wu *et al*. [38].

### Genome sequencing and assembly

The genome was sequenced using a combination of Sanger and 454 sequencing platforms. All general aspects of library construction and sequencing performed at the JGI can be found at http://www.jgi.doe.gov/. 454 Pyrosequencing reads were assembled using the Newbler assembler version 1.1.02.15 (Roche). Large Newbler contigs were broken into 1,716 overlapping fragments of 1,000 bp and entered into assembly as pseudo-reads. The sequences were assigned quality scores based on Newbler consensus q-scores with modifications to account for overlap redundancy and to adjust inflated q-scores. A hybrid 454/Sanger assembly was made using the parallel phrap assembler (High Performance Software, LLC). Possible mis-assemblies were corrected with Dupfinisher [39] or transposon bombing of bridging clones (Epicentre Biotechnologies, Madison, WI). Gaps between contigs were closed by editing in Consed, custom primer walk or PCR amplification. A total of 1,082 Sanger finishing reads were produced to close gaps, to resolve repetitive regions, and to raise the quality of the finished sequence. The error rate of the completed genome sequence is less than 1 in 100,000. Together all sequence types provided 51.2 × coverage of the genome. The final assembly contains 16,169 Sanger and 445,271 pyrosequence reads.

### Genome annotation

Genes were identified using Prodigal [40] as part of the Oak Ridge National Laboratory genome annotation pipeline, followed by a round of manual curation using the JGI GenePRIMP pipeline (http://geneprimp.jgi-psf.org) [41]. The predicted CDSs were translated and used to search the National Center for Biotechnology Information (NCBI) nonredundant database, UniProt, TIGRFam, Pfam, PRIAM, KEGG, COG, and InterPro databases. Additional gene prediction analysis and functional annotation were performed within the Integrated Microbial Genomes Expert Review (IMG-ER) platform (http://img.jgi.doe.gov/er) [42].

## Genome properties

**Figure 3:**
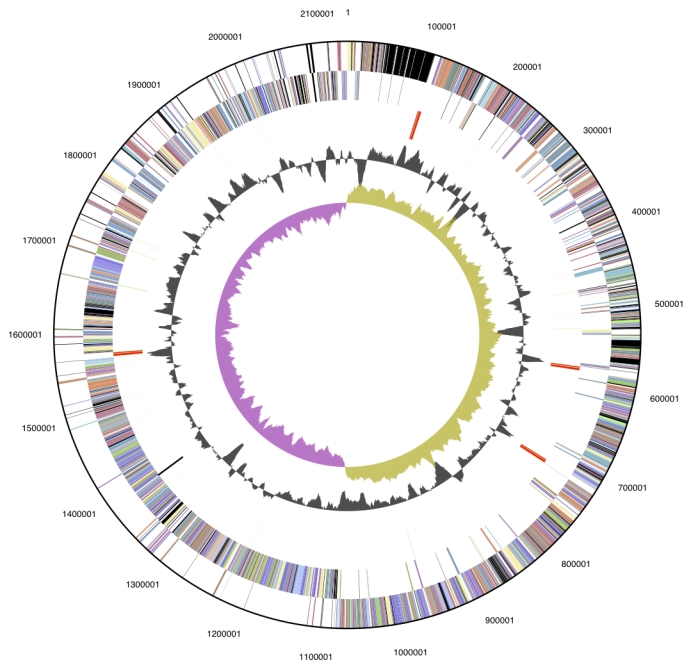
Graphical circular map of the genome. From outside to the center: Genes on forward strand (color by COG categories), Genes on reverse strand (color by COG categories), RNA genes (tRNAs green, rRNAs red, other RNAs black), GC content, GC skew.

The genome is 2,132,142 bp long and comprises one main circular chromosome with a 38.6% GC content (Table 3 and Figure 3). Of the 1,920 genes predicted, 1,859 were protein coding genes, and 61 RNAs; 15 pseudogenes were also identified. The majority (73.6%) of the genes were assigned a putative function while those remaining were annotated as hypothetical proteins. The distribution of genes into COGs functional categories is presented in Table 4.

**Table 3:** Genome Statistics

**Attribute**	Value	% of Total
Genome size (bp)	2,132,142	100.00%
DNA coding region (bp)	1,886,054	88.46%
DNA G+C content (bp)	823,631	38.63%
Number of replicons	1	
Extrachromosomal elements	0	
Total genes	1,920	100.00%
RNA genes	61	3.18%
rRNA operons	4	
Protein-coding genes	1,859	96.82%
Pseudo genes	15	0.78%
Genes with function prediction	1,413	73.59%
Genes in paralog clusters	174	9.06%
Genes assigned to COGs	1,474	76.77%
Genes assigned Pfam domains	1,490	77.06%
Genes with signal peptides	306	15.94%
Genes with transmembrane helices	492	25.62%
CRISPR repeats	5	

**Table 4:** Number of genes associated with the general COG functional categories

**Code**	**value**	**%age**	**Description**
J	142	7.6	Translation, ribosomal structure and biogenesis
A	0	0.0	RNA processing and modification
K	71	3.8	Transcription
L	92	4.9	Replication, recombination and repair
B	2	0.1	Chromatin structure and dynamics
D	20	1.1	Cell cycle control, mitosis and meiosis
Y	0	0.0	Nuclear structure
V	26	1.4	Defense mechanisms
T	29	1.6	Signal transduction mechanisms
M	106	5.7	Cell wall/membrane biogenesis
N	6	0.3	Cell motility
Z	0	0.0	Cytoskeleton
W	0	0.0	Extracellular structures
U	35	1.9	Intracellular trafficking and secretion
O	48	2.6	Posttranslational modification, protein turnover, chaperones
C	100	5.4	Energy production and conversion
G	68	3.6	Carbohydrate transport and metabolism
E	160	8.6	Amino acid transport and metabolism
F	63	3.4	Nucleotide transport and metabolism
H	117	6.3	Coenzyme transport and metabolism
I	33	1.8	Lipid transport and metabolism
P	124	6.7	Inorganic ion transport and metabolism
Q	19	1.8	Secondary metabolites biosynthesis, transport and catabolism
R	177	9.5	General function prediction only
S	143	7.7	Function unknown
-	385	20.7	Not in COGs
